# Utilization of Extraction Procedures for Evaluating Environmental Risk from Waste Materials

**DOI:** 10.3390/toxics11080678

**Published:** 2023-08-07

**Authors:** Dagmar Remeteiová, Silvia Ružičková, Mária Heželová, Ľubomír Pikna

**Affiliations:** Institute of Recycling Technologies, Faculty of Materials, Metallurgy and Recycling, Technical University of Košice, Letná 9, 04200 Košice, Slovakia; dagmar.remeteiova@tuke.sk (D.R.); maria.hezelova@tuke.sk (M.H.); lubomir.pikna@tuke.sk (Ľ.P.)

**Keywords:** ore processing waste tailings, waste materials, extraction procedures, mobility of heavy metals, environmental risk

## Abstract

Several procedures for extracting content from different waste materials types were investigated, with the aim of evaluating their environmental impact. The waste materials consisted of wastes from bauxite ore processing by means of the Bayer process (red mud, Ajka, Hungary), bauxite ore using the sintering process followed by the Bayer process (brown-red mud, Žiar nad Hronom, Banská Bystrica region, Slovakia) and sulphide ores (metal-rich post-flotation tailing, Lintich, Slovakia). The extraction procedures were carried out with the aim of isolating “mobilizable” fractions using 0.05 M ethylenediaminetetraacetic acid (EDTA) and 0.43 M acetic acid (AA) (representing environmental risk during changes in normal environmental conditions) and “maximum potentially mobilizable” fractions using 2 M HNO_3_ (representing the total environmental risk). The content of chosen toxic heavy metals (THMs) (Cd, Cr, Cu, Pb, Ni, Zn) and Fe, Mn as metals creating Fe/Mn oxides in the extracts and solutions after microwave digestion was determined using high-resolution continuum source flame atomic absorption spectrometry (HR CS FAAS). On the basis of the results obtained in this study, it is possible to state that different origin of waste materials is reflected in different mobility of toxic heavy metals into the surrounding environment. From the point of view of toxic heavy metals mobility, disposal site of wastes after bauxite processing are much less of a threat to the environment than disposal site of flotation sludge after processing sulphide ores. The single extraction of 0.43 M AA is more effective than the extraction of 0.05 M EDTA for the purposes of determining the content of metals in the mobilizable fraction of tailing waste materials. The mobility of the studied toxic heavy metals in the Lintich tailing decreases in the direction from the lagoon to the dam, which may indicate the fact that the dam serves to a certain extent to inhibit the mobility of metals into the surrounding ecosystem.

## 1. Introduction

Mining, ore/mineral processing and metallurgical processes generate the following main categories of waste: (I) waste rock from surface or underground mining, (II) tailings as waste from ore processing plants, (III) slag as waste from smelters [1]. For mine waste disposal, common techniques usually involve waste storage facilities (conventional tailings lagoons, pasty and thickened tailings and tailings dumps and piles). Tailings are normally stored under water to prevent the formation of surface dusts and of acid mine drainage in the case of sulphide material presence [2]. After disposal of these solid wastes, secondary wastes such as heap leachates and acid mine water may be generated [3]. Failures of tailings lagoons leading to spills of deposited waste, seepage from unreclaimed sites and direct discharges to waterways can have serious and long-term environmental and social consequences [4]. The chemical composition of tailings depends on the processed ore mineralogy, the nature of the liquid used to extract the usable metals, efficiency of the extraction process and the degree of weathering during storage in the lagoon [2]. The presence of quartz, sulphides and silicates compounds [5] is typical for all types of tailings and, together with oxygen, are usually the most abundant elements [2,5]. The main components often include Al, Ca, K, Mg, Mn, Na, P, Ti and S. Besides these major elements, the wastes also contain toxic heavy metals, which are persistent pollutants in the surrounding environment and are toxic even at low concentrations [1], and pose risks to human health, phytotoxicity, ecotoxicity and water and soil contamination [6]. Other sources of heavy metals in mining areas are liquid wastes, gaseous and dust emissions [3]. Depending on the ore processing method and the exposure of wastes to surface conditions, the decomposition of original minerals may lead to the release of elements from their bonds and their transition into surrounding ecosystems [4], causing the deterioration of groundwater and surface water by leaching [7]. Precisely because these elements did not interact with the overlying ecosystems before mining, they can pose serious problems for the post-mining environment [4].

Brown mud (BM) or red mud (RM), depending on the production method, consists of insoluble residues generated during the processing of bauxite by the aluminium industry [8]. Red mud is the fine fraction of residue produced during bauxite processing with concentrated NaOH using the Bayer process [9,10,11]. The use of NaOH means that red mud is very caustic material with pH 10–13 [9,10,11,12,13,14], as well as with high salinity and conductivity [9]. The composition of red mud depends on the particular ore used, and the typical components are iron oxides, quartz, sodium alumina-silicates, titanium dioxide, calcium carbonate/aluminate and sodium hydroxide [9,12]. It often contains muscovite and crystalline or amorphous silica [8]. Red mud can also contain increased concentrations of potentially toxic or environmental dangerous metals and metalloids, including Pb [12], Cu, Ni and Zn [9], which are predominantly associated with weakly soluble minerals. In the environment, the mobility of Cu and Ni is higher under acidic conditions, whereas in neutral and alkaline conditions they adsorb strongly to minerals and form strong complexes with organic material, too. At present, around 150 million tonnes of red mud is produced globally per year, of which approximately 97–98% is unexploited waste [15]. Red mud is typically stored in large lagoons or in land-based disposal pits [16]. Approximately 50 million tons of red mud is stored in Hungary in lagoons with high-volume storage. In the fall of 2010, the western dam of the red mud reservoir near an alumina plant at Ajka (Hungary) collapsed, causing an environmental disaster [11,16]. The total content of metals in red muds varies considerably, reflecting the different sources of the mud, the methods used for determination and ways of digestion of samples. For the red mud in Hungary, contents of metals varied from 193 to 864 mg/kg for Cr, from 49 to 183 mg/kg for Cu, from 8.2 to 215 mg/kg for Pb, from 26 to 361 mg/kg for Ni and from 78 to 334 mg/kg for Zn [11].

Brown mud (unlike red mud) contains lower proportions of Fe_2_O_3_ [8] and Na_2_O [17]. The brown mud from alumina production near the town of Žiar nad Hronom (ZSNP a.s., Banská Bystrica region, Slovakia) contains 30–35% Fe_2_O_3_, 3–4% TiO_2_, 10–12% Al_2_O_3_, 24–26% CaO, 3–6% Na_2_O, 10 mg/kg Hg, 220 mg/kg Cu, 400 mg/kg Cr, 700 mg/kg V, 150 mg/kg Pb and 800 mg/kg As [8]. The dominant components of this brown mud are iron oxides in the form of crystalline hematite (Fe_2_O_3_) or goethite (FeO(OH)) and alumina in the form of boehmite (γ-AlOOH) [17]. From the environmental point of view, both red and brown mud represent hazardous waste forming a significant environmental burden.

When waste materials from the mining of sulphide ores (waste rocks) and their processing (post-flotation tailings) are exposed to atmospheric oxygen, the result is oxidation of S^2−^ to SO_4_^2−^ and formation of acid mine drainage (AMD) with low pH and high concentrations of sulphates, heavy metals and metalloids [5,6,18,19,20]. Acidification occurs due to the absence of carbonate minerals, which are able to neutralize the acidity generated by the oxidation of sulphides by consuming hydrogen ions [20]. Under acid pH conditions, many toxic metals and metalloids are generally more soluble than in near-neutral pH conditions. Metals leaching through AMD can lead to contamination of the surface and subsurface environment, not only in the adjacent ground, but also often over much more extended areas [6,21]. The rate of metals leaching depends on the chemical and physical properties of the tailings and the prevailing environmental conditions [6]. The process of enrichment of leachate with pollutants/heavy metals from tailings occurs in two phases [22]. Firstly, it is the selective detachment of the pollutants dissolved in the pore water (ionized metals and dissolved complexes) from the basic material. Secondly, when rainwater reaches the surrounding soil, some metals are dissolved, and they become part of the solution, while others remain adsorbed and/or precipitated and move with the solid particles.

Pollution involving heavy metals is a serious problem due to their toxicity, bioavailability/mobility and non-biodegradability in the environment [23]. The bioavailability/mobility of heavy metals in relation to other solid components of the environment depends on their association with phases of solid waste and chemical forms [24,25], occurrence, abundance, reactivity and hydrology [26] and is directly influenced by waste properties (e.g., metal source, pH, redox potential, solubility of organic matter and mineral phases) [27,28,29]. The assessment of environmental and health risks from solid tailings cannot be solved only by means of classical chemical and mineralogical analyses [1]. Knowledge about the mobility, bioavailability and thus potential toxicity of heavy metals is more important for this assessment than their total concentration [25]. Heavy metals, depending on the surrounding material, are bound to the solid substrates in various ways: adsorbed on clay surfaces, or iron and manganese oxyhydroxides, present in the lattice of residual primary mineral phases (e.g., silicates), secondary mineral phases (e.g., carbonates, sulphates and oxides), associated with amorphous materials or complexed with organic matter and also water-soluble extractable compounds [24,29].

Fractionation analysis is a method of analytical chemistry, which enables isolation (fractionation) and quantification (analysis) of different element forms (fractions) according to their various physical or chemical properties (e.g., solubility or chemical bonding) [30,31]. The isolation of fractions by means of extraction is based on the different solubility of element forms in specific defined extraction reagents, which may be applied in a single step or several sequential ordered steps [31]. Both single and sequential extraction methods are important in studying where environmental pollutants end up [31,32]. The main disadvantages of sequential extraction procedures are labor and time intensiveness, nonselectivity of reagents and readsorption phenomena [33]. Single extractions provide fast, cheap and simple assessment methods for monitoring heavy metal mobility in contaminated soils under environmental conditions given by the composition of soil pore water [34,35], sediments [29,33,34,36], and also in pyrometallurgical slags [1], flotation tailings and hydrometallurgical wastes. The different fractionation extractions are often applied to simulate processes in the environment, such as acidification or oxidation [33]. The extracting reagents can be divided into three categories, depending on their nature:

- non-buffered neutral salt solutions, such as CaCl_2_, NaNO_3_, Ca(NO_3_)_2_ or acetic acid [36], in order to extract cations adsorbed onto solid materials, due to permanent structural charges [29],
- buffered complexing/chelating or reducing agents, such as ethylenediamine tetraacetic acid (EDTA) [29,36],
- strong mineral acids with various pH (aqua regia, nitric acid or hydrochloric acid) in order to simulate the effect of acid input (e.g., through acid rain) [1,6,29,34,36,37].

Within the framework of harmonization of fractionation extraction procedures for risk evaluation of heavy metals in soils and sediments, the Standards Measurements and Testing Program (SM&T) of the European Commission implemented an extensive collaborative study, which resulted in the choice of 0.43 M acetic acid (AA) for extraction of “mobilizable/potential available fractions” to estimate the influence of acidification on element mobility [38] and 0.05 M EDTA for extraction of metals from the exchange sites of both organic and inorganic complexes in soils or sediments [39]. The proportion of metals in fractions which are mobile under slight changes in the pH of soils/sediments will be extracted by means of AA and the fraction released from non-silicate phases (inorganic and/or organic) will be extracted by means of EDTA [36]. Extraction by means of strong acids such as nitric or hydrochloric acid, which do not dissolve the silicate matrix, can be used for isolation of the “maximum potentially mobilizable” elements fraction/pseudototal content, which may be mobile during extreme changes in environmental conditions [32]. This extraction method is a useful tool in the assessment of long-term potential risk of toxic elements. It is assumed that single extraction methods enable fractions with differing mobility and bioavailability to be isolated [40]. Most studies focusing on monitoring toxic heavy metals mobility from waste materials, e.g., tailings after processing of sulphide ores [3,7,41,42] or bauxite ore (red mud) [13,43,44,45], use sequential extractions, which were originally developed for natural sediments. The application of less material- and time-consuming single extractions can be a simpler and faster alternative for monitoring the pollution of surrounding soils by toxic heavy metals from tailing storage facilities.

In this paper, we present the results of fractionation of various waste materials using reagents with varying extraction ability: (a) 0.05 M EDTA, (b) 0.43 M acetic acid (weaker extraction ability), and (c) 2 M HNO_3_ (stronger extraction ability). These extraction procedures were applied to solid waste materials representing a range of environmental risks from tailings after: (I) bauxite processing by means of the Bayer process with the highest alkaline character (Ajka, Hungary), (II) bauxite processing by means of the sintering process with lower alkaline character (Žiar nad Hronom, Slovakia), and (III) sulphide ore processing with acidic character (Lintich, Slovakia).

The main goals of the experiments were: (1) to evaluate the total load of selected types of waste materials with heavy metals, (2) to test the suitability of EDTA and AA for the isolation of the “mobilizable” fraction of toxic heavy metals from waste materials after the processing of mineral raw materials deposited in tailings for rapid monitoring of their possible impact on the surrounding ecosystem, and (3) to evaluate the impact of the different nature of the deposited material on the mobility of heavy metals from tailings. 

The use of single extraction represents a new approach in the environmental risk assessment, which can be very useful in the prediction of the state of the environment near the stress areas, or in the case of an environmental accident. It is a less material- and time-consuming method that provides data with great informative value. 

## 2. Materials and Methods

### 2.1. Studied Site Description

**Figure 1:**
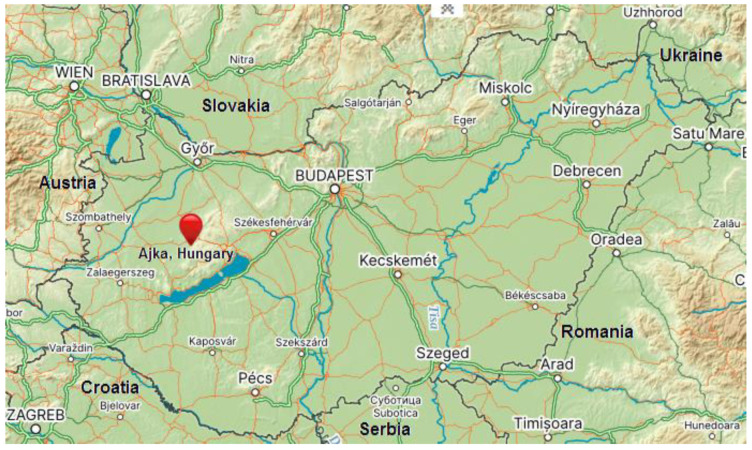
Study area Ajka (Hungary) [46].

In Hungary, intensive bauxite mining and processing using the Bayer process was carried out between 1940 and 1990 [14]. The red mud as a waste product of this bauxite treatment was deposited in a tailing dam at Ajka in western Hungary (Figure 1) using the wet technique on the land surface with additional construction of a dam. On 4 October 2010, after a breach of the tailing dam, about one million cubic meters of highly alkaline (pH ≈ 13) red mud from one of the Ajka reservoirs spilled out into the surrounding area [10,12,14,15].

**Figure 2:**
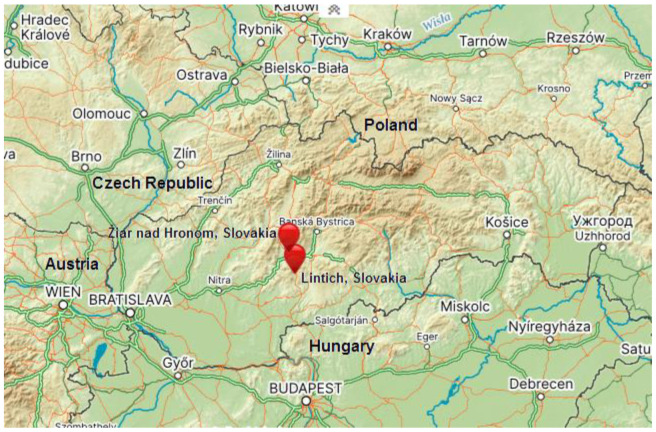
Study areas Žiar nad Hronom and Lintich (Slovakia) [46].

Near the town of Žiar nad Hronom in central Slovakia (Figure 2), processing of bauxite was done predominantly by means of the sintering process in the years 1957–1997. The bauxite ore was extracted with sodium hydroxide, and the residue (brown mud) was stored in a reservoir [8]. The sludge field has a length of approx. 1000 m, the width across the middle is approx. 460 m and height 42 to 45 m above the surrounding land, with a total area of 44.68 ha [17]. The reservoir contains a total of about 10 million tons of brown mud (pH in the range of 9–13 and high ionic strength), consisting of an insoluble mixture of several compounds originally present in the bauxite, and of compounds formed or introduced during its processing [47].

In the last century, Slovakia was an important producer of various metals and metalloids (Ag, Au, Cu, Fe, Hg, Sb and Zn), which were associated mainly with sulphide ores [20]. The wastes after flotation processing of ores were deposited in tailings impoundments. The tailing dam at Lintich, part of Banská Štiavnica town in central Slovakia (Figure 2), contains 580,000 m^3^ of fine-grained materials from flotation processing of sulphide ores [19]. The studied tailing containment originated from hydrotechnical works as an artificial water reservoir, and the locality is included in the current register of confirmed environmental burdens. Despite the fact that the Lintich containment was shut down in 1975, there are still high concentrations of heavy metals in the area. Over time, the sediments changed into clayey-silty sands with high porosity and containing significant amounts of water. The surface of the tailing containment is predominantly covered with secondary minerals (gypsum) [48] and a thin, irregular layer of humus [19].

### 2.2. Waste Materials Sampling and Analysis

Samples of acidic waste materials from the post-flotation tailing dam at Lintich were collected from probe bores excavated in the area of contact between the beach and the surface of the lagoon (L1) and from the wall of the tailing dam beside the containment (L2). Samples of alkaline waste materials from the tailing dam at Ajka (A1, red mud) were collected from the dam wall, and from the tailing dam at Žiar nad Hronom (ZH2, previously brown mud) also from the dam. The samples were collected by means of single sampling from depth approx. 20 cm. After collection, all final samples of waste materials were first dried at room temperature; the compacted particles were separated by rubbing in an agate bowl and then sieved through a 0.125 mm sieve.

Single extraction procedures were carried out by applying the following extraction reagents and conditions:

- Extraction with EDTA: a mixture of 5 g dry waste material and 50 mL 0.05 M EDTA (buffered, pH = 7) was shaken for 1 h in a polyethylene vessel at room temperature [39,49]. This extraction reagent isolates the EDTA “mobilizable” fraction.
- Extraction with acetic acid (AA): a mixture of 1 g dry waste material and 40 mL 0.43 M AA (non-buffered, pH = 3) was shaken for 16 h in a polyethylene vessel at room temperature [38,49]. This extraction reagent isolates the AA “mobilizable” fraction.
- Extraction with nitric acid: a mixture of 5 g dry waste material and 50 mL 2 M HNO_3_ (non-buffered, pH = 0.7) was shaken for 6 h in a polyethylene vessel at room temperature. This extraction reagent isolates the “maximally potentially mobilizable” fraction [50].

Applied extraction procedures were selected on the basis that they are validated procedures for EDTA [39,49] and AA extraction [38,49], and in the case of HNO_3,_ using it is a commonly used procedure in Slovakia for potentially releasable contents of toxic heavy metals [50].

The extraction procedures were carried out with mechanical shaking (equipment: laboratory shaker MRC TS–400 D, M.R.C. Ltd., Hagavish, Israel) of a suspension of the sample and the extraction reagent in a 100 mL polyethylene extraction vessel at laboratory temperature and 240 rpm. After the extractions were completed, the suspensions were passed through filter paper with narrow pores (blue ribbon) and paper diameter = 18.5 cm. All vessels were cleaned by washing in 4 M nitric acid, thereafter with an extraction reagent, and rinsing with deionized water [49]. The total elements content in the samples was determined after microwave wet acid digestion (equipment: Ethos One, Milestone, Milan, Italy), which represents decomposition of samples using concentrated acids and microwave radiation. The samples of waste materials from Lintich (0.3 g) were digested with a mixture of 2 mL HNO_3_ + 6 mL HCl + 2 mL HF, and the samples of waste materials from Ajka and Žiar nad Hronom (0.3 g) were digested with a mixture of 6 mL HNO_3_ + 2 mL HCl + 2 mL HF at temperature 210 °C for 15 min. The contents of chosen elements in each extract and in the solutions after digestion were determined using the high-resolution continuum source flame atomic absorption spectrometry (HR CS FAAS) method (equipment: contrAA700, Analytik Jena, Jena, Germany). Each decomposition and extraction experiment was performed in three repetitions.

## 3. Results and Discussion

### 3.1. Determination of Total Contents

The total content of chosen elements after wet acid digestion of waste material samples was determined. The results of total elements content determination in the studied waste materials converted to mg/kg are listed in Table 1.

The results in Table 1 show that all chosen metals, except Fe, occur with higher content in the waste material from the Lintich containment with acidic character of the material. The dominant element in alkaline waste materials A1 and ZH2 was Fe as a component of iron oxides [8,9,12,17]. The total content of chosen elements in the acidic waste materials from Lintich L1 and L2 decreased in this order: Fe (major element), Mn, Zn, Pb, Cu (minor elements), Cd (trace element). The determined total contents of minor elements are comparable to the average composition of the flotation sludge in the Lintich tailing dam [51]. The elements Cr and Ni in these waste materials were not detected with the HR CS FAAS method used. Their contents were below limit of detection (LOD): LOD_Cr_ 0.05 mg/L; LOD_Ni_ 0.04 mg/L. The content of Fe, Mn, Pb, Cu decreased in the direction from the lagoon to the dam, and the content of Zn and Cd increased in the same direction. This phenomenon is probably related to the different origin of the material in the lagoon (waste material) and wall of the tailing dam, which was artificially created. In the strong alkaline waste material from Ajka A1, the total elements content decreased in this order: Fe (major element), Mn, Cr, Zn, Ni (minor elements), Pb, Cu (trace elements), while Cd was not detected in this sample (LOD_Cd_ 0.015 mg/L). Similar contents, except for Cr, or the trend of decreasing contents was also found in [13]. The total elements content in the waste material from Žiar nad Hronom ZH2 (less alkaline than A1) decreased in this order: Fe (major element), Mn, Zn, Cr, Ni (minor elements), Pb, Cu (trace elements), and the presence of Cd was not confirmed. In the sample A1 there was higher content of Fe, Cr and lower content of Mn, Zn, Pb and Cu than in the sample ZH2. 

### 3.2. Determination of Element Contents in the Mobilizable Fractions

For isolation of the “mobilizable” elements fraction, two extraction reagents were used: AA (weak organic acid) and EDTA (organic chelating agent). The extracting effect of these reagents depends on the elements ability to associate with phases of the waste material sample matrix and on its composition. The content of elements in the “mobilizable” fraction represents the “mobile” and “potentially mobile” fractions, which may be mobile during moderate changes in normal environmental conditions, caused for example by acidification of the environment. The content of elements in the EDTA “mobilizable” fraction (EDTAMF) in mg/kg is listed in Table 2 and in the AA “mobilizable” fraction (AAMF) in Table 3. 

The results listed in Table 2 and Table 3 show differences between the elements content in “mobilizable” fractions depending on the extraction reagent used, the type of waste material and the specific element. Although the total content of Zn and Cd in the Lintich tailings dam is higher than in the lagoon itself (Table 1), their content in both EDTAMF and AAMF is lower in the dam than in the lagoon. Contents of Pb and Cu in EDTAMF and AAMF as well as the total contents (Table 1) decreased from the lagoon towards the dam. These findings suggest that the tailings dam itself may significantly promote the mobility of chosen toxic heavy metals in the direction of the surrounding soil ecosystems, and it is also possible to assume higher mobility of Pb and Cu from lagoon to groundwater than to the dam. The content of toxic heavy metals mobilizable by EDTA and AA from alkaline waste materials A1 and ZH2 was significantly lower than from the acidic waste materials of the Lintich tailings. The content of elements in EDTAMF from ZH2 was lower than from A1, despite the higher total content of these elements in ZH2 than in A1. The content of elements in AAMF from ZH2 was higher than from A1, except for Pb. 

The mobility of elements is dependent on the phase composition of the waste materials matrix and the elements’ ability to associate with these phases. It can be assumed that, if the phase composition of the waste materials matrix in the monitored area does not change significantly, an increase or decrease in the total content of the element will also lead to an increase or decrease in its content in the monitored fraction. It is more appropriate to use the percentage of the element in the fraction with respect to its total content in the waste material to express the mobility of the element. The percentage mobility of the element also represents the percentage recovery of the element in the extract or extraction efficiency of the used extractant, respectively. The percentage recovery (R) was calculated according to the Equation (1):

(1) $$R\left(\%\right)=\frac{c_{Me,F}}{c_{Me,T}}\times 100$$

where $c_{Me,F}$ is content of the metal in the fraction (mg/kg), $c_{Me,T}$ is total content of the metal in the sample (mg/kg).

**Figure 3:**
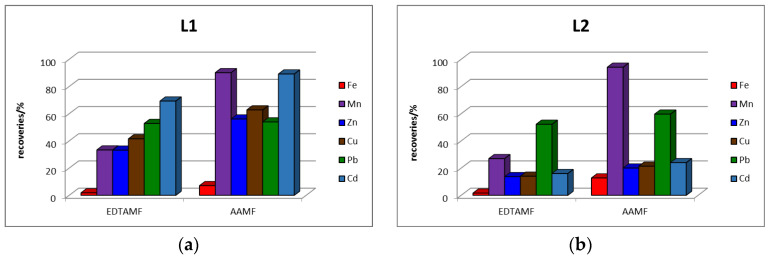
Percentage recoveries of elements in extracts of EDTAMF and AAMF from: (**a**) L1 and (**b**) L2.

**Figure 4:**
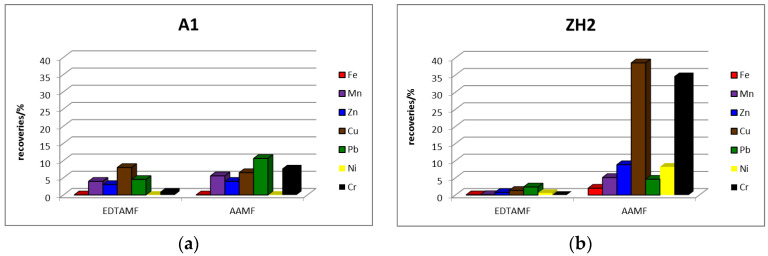
Percentage recoveries of elements in extracts of EDTAMF and AAMF from: (**a**) A1 and (**b**) ZH2.

Comparison of the percentage recoveries of elements in the EDTA and AA extracts (percentage mobility) from acidic waste materials is presented in Figure 3 and from alkaline waste materials in Figure 4. 

The graphs in Figure 3 show that the percentage shares of Zn, Cu and Cd in both monitored “mobilizable” fractions are higher in the waste material originating from the Lintich tailings lagoon (L1) than in the waste material originating from the dam of this containment (L2). From these results it is possible to state that the content of Zn, Cu and Cd in the “mobilizable” fraction decreased in the direction from lagoon to dam. The mobility of Mn and Pb was approximately similar in lagoon and dam. The mobility of Fe was very low in both waste materials, which suggests that Fe is present in acidic waste materials in forms insoluble in the extraction reagents used. For isolation of the “mobilizable” elements fraction from waste material with acidic character of deposited material, it is preferable to use AA, which has higher extraction efficiency than EDTA. The percentage contents of Cd, Zn and Pb in the AAMF of the Lintich tailing detected in our study are comparable to the contents in the fraction isolated in the 1st step of the BCR and in the sum of the contents corresponding to the fraction isolated in the 1st and 2nd steps of the Tessier sequential extraction of tailings material from the flotation processing of sulphide ores found in the frame of other authors’ study [3]. The slightly higher content of Pb and Zn detected by the application of single extraction is related to the use of 0.43 M AA for extraction opposite to 0.11 M AA, which is used in sequential extractions. 

The graphs in Figure 4 show that the percentage content of elements in EDTAMF appears higher in the A1 sample than in the ZH2. The percentage share of Fe, Ni and Cr in EDTAMF from both waste materials is very low (less than 1%), which suggests that these elements are present in the alkaline waste materials in EDTA insoluble forms. The percentage share of elements in the AA “mobilizable” fraction, except for Mn and Pb, is higher in the ZH2 sample than in the A1. The higher extraction efficiency for isolation of the “mobilizable” fraction from both alkaline waste materials was observed from the application of AA, similar to the results from acidic waste materials. This phenomenon is probably related both to the ability of the elements to associate with those phases of the material that are more extractable into AA than into EDTA, and to the pH value, or higher acidity of AA than EDTA, respectively.

### 3.3. Determination of the Element Contents in the Maximum Potentially Mobilizable Fractions

**Figure 5:**
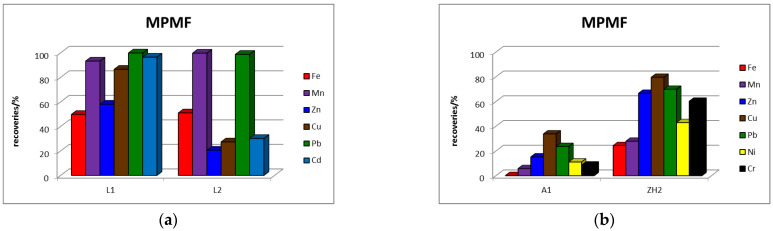
Percentage recoveries of elements in the extract of MPMF in: (**a**) acidic waste materials and (**b**) alkaline waste materials.

For isolation of the “maximally potentially mobilizable” fraction (MPMF), 2 M HNO_3_ (strong inorganic acid) was used, and the content of elements in the HNO_3_ extract represents long-term potential environmental risk. The elements content (mg/kg) in the MPMF is numerically expressed in Table 4, and Figure 5 graphically typifies the percentage elements shares in MPMF for the samples L1 and L2 (Figure 5a), A1 and ZH2 (Figure 5b).

Utilization of 2 M HNO_3_ for the extraction led to release of approx. 50% Fe and 95% Mn of their total content in the L1 and L2 samples (Figure 5a), which is significantly higher than from the A1 (approx. 0.2% Fe and 6% Mn) and ZH2 (approx. 25% Fe and 28% Mn) samples (Figure 5b). These results may indicate that in alkaline waste materials, a significant share of Fe and Mn is associated with their silicate matrix. Despite the lower percentage recoveries of Fe and Mn in the 2 M HNO_3_ extract, the absolute amounts of these elements are high (Table 4). 

### 3.4. Evaluation of the Mobility of Elements in the Chosen Waste Materials

The percentage recoveries of Fe, Mn and Pb in the MPMF from L1 and L2 were approximately similar (Figure 5a), but the absolute contents of these elements in mg released from 1 kg of waste materials were different (Table 3) and decreased in this order: Fe, Mn, Pb. The contents of Zn, Cu and Cd in % weight (Figure 5a) as well as in mg/kg (Table 4) in MPMF were higher in the L1 than in the L2 sample. The percentage contents as well as absolute contents in mg/kg of all chosen elements in MPMF from the ZH2 sample were higher than from the A1 sample (Figure 5b), which suggests that the mobility of these elements during extreme changes in conditions (environmental accident) may be greater from the ZH2 (less alkaline waste material) than from the A1 sample (strong alkaline waste material).

**Figure 6:**
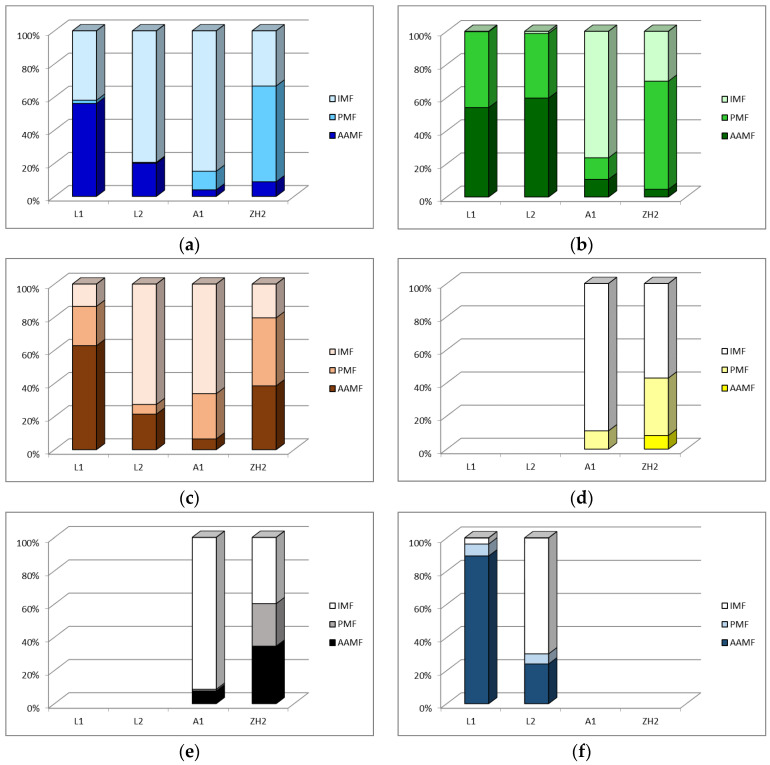
Percentage share of toxic heavy metals: (**a**) Zn, (**b**) Pb, (**c**) Cu, (**d**) Ni, (**e**) Cr and (**f**) Cd in the AA “mobilizable” fraction, “potentially mobilizable” fraction and “immobile” fraction.

Figure 6 graphically shows the mobility of the individual toxic heavy metals in the studied waste materials expressed as the element percentage share in:

- the AAMF (fraction with higher percentage share of elements than EDTAMF),
- the “potentially mobilizable” fraction (PMF, difference between percentage shares of an element in MPMF and AAMF),
- the “immobile” fraction (IMF, difference between total element content, i.e., 100%, and the percentage share in MPMF).

The percentage share of an element in PMF represents element forms associated predominantly with Fe/Mn oxides and/or sulphides, while the share in IMF (residual fraction) represents element forms associated with the silicate matrix [31].

The highest mobility of chosen toxic heavy metals in AAMF and PMF (Figure 6) was found in the L1 sample (the lagoon of Lintich tailing dam). The mobility of elements in the L2 sample (the dam of this containment), except for Pb, was lower than in the L1 sample. The highest mobility of elements in PMF was found for the ZH2 sample, and the highest mobility in AAMF for the L1 sample. The mobility of elements in AAMF and PMF from the ZH2 sample was higher than from the A1 sample, except for Pb in AAMF. Despite the fact that RM from A1 can be in terms of higher alkalinity more dangerous for the surrounding ecosystem than BM from HZ2, from the point of view of possible toxic heavy metals mobility, the opposite is true. The ability of RM to bind heavy metals in the immobile fraction is also often used to reduce their mobility in soils by adding RM to the soil [11,16]. From all chosen toxic heavy metals, the highest mobility was determined for Pb (Figure 6b). The highest mobility of Pb in tailings material from Lintich (sludge from flotation processing of sulphide ores) is in agreement with the results of applying sequential extraction to similar material [42].

Because the percentage shares of the elements in the PMF may correspond to their association with Fe/Mn oxides or sulphides, the solubility of Fe/Mn phases in 2 M HNO_3_ was evaluated, too. The percentage shares of Fe, Mn and chosen heavy metals in the PMF are expressed in Table 5 and their contents in mg/kg in the PMF in Table 6.

The solubility of Fe/Mn phases from the strong alkaline A1 sample expressed in % weight (Table 5) and mg/kg (Table 6) is very low. Despite the fact that the total Fe content in the ZH2 is lower than in the A1 sample (Table 1), the solubility of Fe phases from the ZH2 is markedly higher than from the A1 sample (Table 6), which also leads to the highest percentage mobility of chosen toxic heavy metals in the PMF from the ZH2 sample (Table 5). The solubility of Fe/Mn phases from acidic waste materials (L1 and L2) is approximately similar (Table 5), but the mobility of toxic heavy metals in the PMF is higher from the L1 (lagoon) than from the L2 sample (dam).

The percentage that selected risk elements share in the IMF corresponding to the content of elements, which will not move from tailing containments to the surrounding environment under any environmental conditions, is given in Table 7.

From Table 7 it follows that the highest percentage shares of elements in the IMF or the residual fraction were determined in the waste material from Ajka (A1, red mud). This fact is in agreement with the results of the application of Tessier’s sequential extraction on red mud from Ajka, which pointed to more than 80% of the toxic heavy metals monitored by us in the residual fraction [13]. Based on this fact, it is possible to state that the chosen toxic heavy metals in this waste material are very strongly bonded in the silicate matrix, which is consistent with the results of other studies [13,43]. The percentage shares of elements in the waste material from Žiar nad Hronom (ZH2, brown mud) are lower than in that from Ajka. The lowest percentage share in the IMF is for Pb in the waste material of the Lintich tailings dam, for which it is also true that in the area of the dam there is an increase in the share of elements in the IMF compared to that in the tailing lagoon.

## 4. Conclusions

The applied extraction procedures enable assessment of the possible mobility of elements within waste materials, and from these to the surrounding environment through the water medium during moderate or marked changes in environmental conditions. From the results obtained in this experimental study, it is possible to assess the environmental impact of Cd, Cu, Cr, Ni, Pb and Zn in the studied waste materials, as follows:

- The extraction efficiency of EDTA and AA for evaluation of elements’ mobility during moderate environmental condition changes are different and depend on the phase composition of the waste material and the elements’ ability to associate with specific phases. For both types of waste materials from tailing containments, either with acidic or alkaline character of the material, it is more efficient to use 16 h extraction with AA (which has higher extraction efficiency) than 1 h extraction with EDTA.
- The dam of the Lintich tailings containment appeared to significantly promote mobility of the chosen toxic heavy metals in the direction of the surrounding soil ecosystems. The content of toxic heavy metals in the EDTA and AA “mobilizable” fractions from alkaline waste materials is significantly lower than from acidic ones.
- Marked changes in the Lintich tailings dam (e.g., environmental accident, extreme decrease in pH) may lead to the release of considerable amounts of Zn and Pb from the waste material (“maximally mobilizable” fraction) into the groundwater. Despite the fact that contents of Zn and Pb in the “maximally mobilizable” fraction in the Lintich tailing waste materials were approximately equal (about 2330 mg/kg Zn and 2310 mg/kg Pb in the lagoon, and about 1130 mg/kg Zn and 1130 mg/kg Pb in the dam), these contents represent different percentage shares released from the total elements content (about 58% Zn and 98% Pb in the lagoon, and about 20% Zn and 98% Pb in the dam). The mobility of the chosen toxic heavy metals during extreme changes in conditions (environmental accident) may be higher from ZH2 (less alkaline material) than from A1 sample (strong alkaline material).
- Waste materials from sulphide ore processing involve much higher risk for the environment than bauxite ore processing waste.
- The chosen toxic heavy metals in the waste material from Ajka (red mud) are predominantly associated with the residual/immobile fraction (silicate matrix), and this waste material can be considered as involving the least risk of heavy metals spreading into the surrounding ecosystem.
- In the frame of research activities, minimal attention is paid to the use of time-, financially- and material-less demanding single extractions for samples of sludge materials. Single extraction of 0.43 M AA appears to be a suitable alternative for rapid screening of similar materials in the field of environmental impact control, in comparison to sequential extractions. If the results of a single extraction show significant differences from long-term trends, it will be necessary, for a deeper study, to use a sequential extraction.
- Single extraction of 2 M HNO_3_ represents a useful tool for rapid prediction of the state and riskiness of the surrounding tailings ecosystem in the case of environmental accident.

## Figures and Tables

**Table 1:** Total elements content (mg/kg) in waste materials.

Element	Fe	Mn	Zn	Cr	Pb	Cu	Ni	Cd
Sample	Content (mg/kg)							
**L1**	35,511	9028	4004	<LoD	2312	613.1	<LoD	14.34
**L2**	23,354	5901	6075	<LoD	1143	355.1	<LoD	24.80
**A1**	452,719	2034	210.0	281.6	69.81	47.96	122.8	<LoD
**ZH2**	263,313	2491	311.8	147.1	76.16	74.73	189.3	<LoD

**Table 2:** EDTA “mobilizable” THMs content (mg/kg) in waste materials.

Element	Zn	Cr	Pb	Cu	Ni	Cd
Sample	Content (mg/kg)					
**L1**	1330	<LoD	1221	254.6	<LoD	9.939
**L2**	832.1	<LoD	596.9	49.79	<LoD	3.969
**A1**	6.330	2.592	3.141	3.823	<LoD	<LoD
**ZH2**	2.355	<LoD	1.790	0.9943	1.106	<LoD

**Table 3:** AA “mobilizable” THMs content (mg/kg) in waste materials.

Element	Zn	Cr	Pb	Cu	Ni	Cd
Sample	Content (mg/kg)					
**L1**	2247	<LoD	1247	385.0	<LoD	12.79
**L2**	1219	<LoD	682.2	76.10	<LoD	5.948
**A1**	8.316	21.60	7.408	3.110	<LoD	<LoD
**ZH2**	27.56	50.92	3.504	28.79	15.560	<LoD

**Table 4:** Elements content in MPMF (mg/kg) of waste materials.

Element	Fe	Mn	Zn	Pb	Cu	Ni	Cr	Cd
Sample	Content (mg/kg)							
**L1**	17,644	8415	2325	2308	530.5	<LoD	<LoD	9.939
**L2**	11,899	5883	1248	1128	96.88	<LoD	<LoD	3.696
**A1**	690.1	117.6	31.92	16.50	16.22	13.56	24.40	<LoD
**ZH2**	64,130	694.8	207.8	53.23	59. 49	81.28	88.61	<LoD

**Table 5:** Percentage elements shares in the PMF from waste materials.

Element	Fe	Mn	Zn	Pb	Cu	Ni	Cr	Cd
Sample	Share (Weight %)							
**L1**	47.87	59.85	24.84	47.02	45.00	<LoD	<LoD	27.09
**L2**	49.32	72.71	6.846	46.51	13.26	<LoD	<LoD	14.10
**A1**	0.142	1.821	12.18	19.14	25.84	11.04	7.743	<LoD
**ZH2**	24.31	27.78	65.90	67.54	78.28	42.36	60.23	<LoD

**Table 6:** Elements content in PMF (mg/kg) from waste materials.

Element	Fe	Mn	Zn	Pb	Cu	Ni	Cr	Cd
Sample	Content (mg/kg)							
**L1**	17,000	5404	994.7	1087	275.9	<LoD	<LoD	3.886
**L2**	11,518	4291	415.9	531.5	47.08	<LoD	<LoD	3.497
**A1**	655.6	37.03	25.59	13.36	12.39	13.56	21.81	<LoD
**ZH2**	64,005	692.1	205.5	51.44	58.50	80.17	88.61	<LoD

**Table 7:** Percentage elements share in the IMF of waste materials.

Element	Zn	Pb	Cu	Ni	Cr	Cd
Sample	Share (Weight %)					
**L1**	41.93	0.1772	13.48	<LoD	<LoD	3.607
**L2**	79.46	1.267	72.72	<LoD	<LoD	69.89
**A1**	84.80	76.36	66.19	88.96	91.34	<LoD
**ZH2**	33.35	30.11	20.39	57.06	39.77	<LoD

## Data Availability

Data will be made available on request.
